# Determinants of an unintended pregnancy among women attending ante natal care at health facilities in Lemi Kura sub city, Addis Ababa, Ethiopia: Institution based case-control study

**DOI:** 10.1371/journal.pgph.0004920

**Published:** 2026-07-09

**Authors:** Amideyesus Adinaw Lopiso, Teketel Tumebo Tirramo, Sisay Tanie, Kaleab Tadesse Worku, Nebiyu Dereje, Tamane Achamo, Andamlak Gizaw Alamdo

**Affiliations:** 1 Research Training Directorate, Armauer Hansen Research Institute, Addis Ababa, Ethiopia; 2 Kind Solution and Consultancy, Africa CDC, Addis Ababa, Ethiopia; 3 Department of Epidemiology, School of Public Health, St. Paul Hospital Millennium Medical College, Addis Ababa, Ethiopia; 4 Wachemo university, College of Medicine and Health Science, Hossaena, Ethiopia; 5 Knowledge Management Division, Africa CDC, Addis Ababa, Ethiopia; 6 Director General Office, Armauer Hansen Research Institute, Addis Ababa, Ethiopia; 7 Department of Health Service Management, School of Public Health, St. Paul Hospital Millennium Medical College, Addis Ababa, Ethiopia; United Nations, UNITED STATES OF AMERICA

## Abstract

Unintended pregnancy is an unplanned or unwanted at conception. It carries significant social, emotional, and health consequences for women, families and communities. In Ethiopia, approximately one-third of married women report that their pregnancies are an unintended. Despite this burden, evidence on contributing factors remains limited, particularly in Lemi Kura sub city. This study, aimed to identify determinants of an unintended pregnancy among women attending antenatal care services in health facilities in Lemi Kura, Addis Ababa. We conducted a facility-based case-control study in Lemi Kura sub-city, Addis Ababa, Ethiopia, from January 3 to February 18, 2022. Using cluster random sampling, 103 cases (women with an unintended pregnancies) and 197 controls (women with planned pregnancies) attending antenatal care units were selected. Data were collected through interviewer-administered, pretested questionnaires. The sample size was calculated using Epi-Info version 7. Analysis was performed in SPSS version 20, applying multivariable logistic regression. Results were reported as adjusted odds ratios (AOR) with 95% confidence intervals (CI), and statistical significance was declared at *p* < 0.05. The Muslim women (AOR: 3.33, 95% CI: 1.21–9.15); Protestant women (AOR: 3.06, 95% CI: 1.07–8.75); reduced women’s autonomy (AOR: 16.58, 95% CI: 2.40–114.15); residing 4 km or more from the antenatal care facility (AOR: 2.70, 95% CI: 1.07–8.75); having two or more living children(parity) (AOR: 5.37, 95% CI: 1.60–18.15); desiring two or fewer children(Ideal) (AOR: 3.63, 95% CI: 1.50–9.01); and having multiple sexual partners (AOR: 4.25, 95% CI: 1.38–30.38) were significant predictors of unintended pregnancy. Low knowledge of family planning methods, unmarried, religion, low autonomy, multiparous, natural method use, health service inaccessibility and having multiple sexual partners predicted unintended pregnancy. For intervention, engaging religious leaders and focusing on multiparous with empowering women is essential.

## Introduction

Unintended pregnancy (UP) is a significant public health concern worldwide, referring to pregnancies that are either mistimed or unwanted at the time of conception. A mistimed pregnancy occurs earlier than desired, typically defined as occurring two or more years before a woman intended to conceive [1], whereas an unwanted pregnancy is one that is not desired at any point in the future [2]. These pregnancies can result in range of outcomes, including abortion, miscarriage, or unplanned birth, each carrying important implications for maternal and child health.

Unintended pregnancies contribute significantly to elevate the risk of morbidity and mortality among mothers, specially in a setting with limited access to maternal healthcare services. When Pregnancies happen at very young or old age or too frequently, likelihood of complications during pregnancy, labor, delivery, and the postpartum period increases [3]. Women experiencing unintended pregnancies face significant health risks, economic burdens, and social consequences, including disruptions in education and employment opportunities [2].

The burden of unintended pregnancy is disproportionately higher in developing countries compared to developed nations [4]. Findings from a systematic review of 29 articles from nine countries performed in may 2019 indicate that Over forty percent of pregnancies worldwide are unintended, with a quarter of these from Africa [5]. Despite international efforts to address the issue, unintended pregnancy rates remain high, particularly in sub-Saharan Africa because of inadequate health system capacity and socio economic inequality [6]. Several socio-demographic factors, including low socioeconomic status, limited access to and inconsistent use of contraceptives, early or late reproductive age, and lower educational attainment, have been identified as key contributors to unintended pregnancies [7]. Additional determinants include parity, age at marriage, media exposure, religious influence and awareness of family planning methods [3]. In Ethiopia, EDHIS 2016 found that 17% of women described their last pregnancy as mistimed, while 8% categorized it as unwanted [8], and despite the availability of family planning services, unintended pregnancy rates have continued to rise in Ethiopia, exacerbating maternal and child health challenges [3].

One of the most major consequence of unintended pregnancy is unsafe abortion, which is associated with serious health complications, including infertility, severe infections, and maternal death [9]. A study conducted by the Guttmacher Institute in Ethiopia reported an incidence of 101 unintended pregnancies per 1,000 women aged 15–44, while approximately 42% of all pregnancies were unintended nationally. In Addis Ababa, the prevalence was even higher, with an estimated 49 unintended pregnancies per 1,000 women [10].

Globally, unintended pregnancies remain a major public health concern. Recent estimates indicate that approximately 121 million unintended pregnancies occur each year, representing about 48% of all pregnancies worldwide. Of these, nearly 61% end in abortion, many of which are unsafe, particularly in low and middle income countries [11].

Globally, about 40% of all unintended pregnancies occur among women under 25 years of age [12]. Each year, an estimated 121 million unintended pregnancies occur worldwide. Unsafe abortions also take a heavy toll—they contribute to roughly 39,000 maternal deaths annually [13,14]. The burden is greatest in developing countries, where maternal and newborn death rates remain persistently high. In sub-Saharan Africa, the maternal mortality ratio is approximately 536 deaths per 100,000 live births. This means a woman’s lifetime risk of dying from pregnancy-related causes is 1 in 38. For comparison, that risk is just 1 in 5,400 in high-income countries [15]. These stark differences underscore the urgent need for targeted interventions to improve access to safe abortion care, post-abortion services, and comprehensive family planning especially in low-resource settings [12].

Women’s autonomy and accessibility to family planning services play a critical role in the prevention of unintended pregnancies. Limited decision-making power, coupled with gaps in contraceptive availability and use, increases women’s vulnerability to unintended pregnancies [16]. In Ethiopia, many women resort to unsafe abortion practices due to unplanned pregnancies, resulting in a range of negative health and social outcomes, including increased risk of maternal mortality, reproductive health complications, economic strain, and psychosocial distress [17].

**Fig 1:**
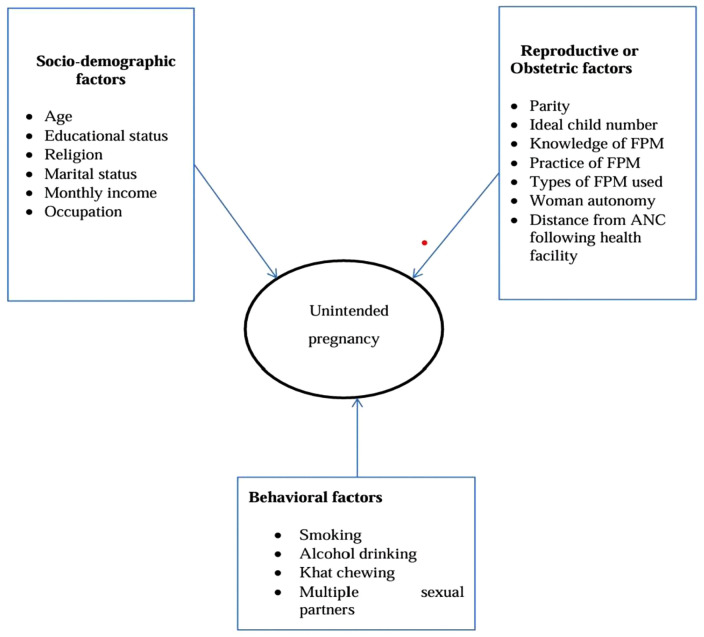
Conceptual framework adopted from different reviewed literature [3,9,16–35].

Lemi Kura sub-city in Addis Ababa, established in 2020, has limited research on the determinants of unintended pregnancy among women attending antenatal care (ANC) services.The absence of documented studies in this newly founded sub-city highlights the need for a case-control study to assess the key determinants of unintended pregnancy in this population. Understanding these factors will provide valuable evidence for policymakers and healthcare providers, facilitating the development of targeted strategies to reduce unintended pregnancies and improve reproductive health outcomes. The findings will also support evidence-based policymaking, strengthen family planning program implementation, and serve as a foundation for future research on reproductive health in Ethiopia (Fig 1).

## Methods and materials

### Study design and setting

An institution-based unmatched case-control study was conducted among pregnant women attending antenatal care (ANC) from January 3,2022 to February 18, 2022, in Lemi Kura Sub-city, Addis Ababa, Ethiopia. Lemi Kura is the newest of the city’s eleven sub-cities, established in 2020, and is bordered by Bole and Yeka sub-cities of Addis Ababa city administration as well as the Oromia Region. In 2022, the sub-city had an estimated population of 344,944, with 165,573 males and 179,371 females. Administratively, it is divided into 10 woredas. According to the 2021 annual report from the sub-city health department, there are 318 health facilities including 126 health service providing clinics and 192 pharmacies (10 public and 308 private). Reproductive health services, including antenatal care (ANC), are offered by 14 health institutions including 4 hospitals, 1 specialty center and 9 health centers, serving an estimated 1,799 ANC visits per month. The study was carried out at one private hospital (ICMC) and three public facilities: Abebech Gobena Hospital, Woreda 13 Health Center, and Hidasse Health Center.

We intentionally included both hospitals and health centers to ensure representation of different levels of health care delivery. Since antenatal care, family planning, and abortion related services are provided across all facility types in Ethiopia, inclusion of both settings improves generalizability and captures diverse population groups.

### Population and sampling

The study population were pregnant women attending antenatal care (ANC) in Lemi Kura Sub-city, Addis Ababa (January 3-February 18, 2022) without restriction on residence or age. A cluster random sampling approach was employed, in which four of the 14 health facilities providing antenatal care service (three government-run, one private) were selected by using a simple random techenique. Cases were women with unintended pregnancies (unwanted or mistimed by < 2 years), while controls were those had planned pregnancies (1:2 ratio). Systematic sampling was applied with in each selected facilities, whereby for every unintended pregnancy case, two consecutive controls with planned pregnancies were included.

**Fig 2:**
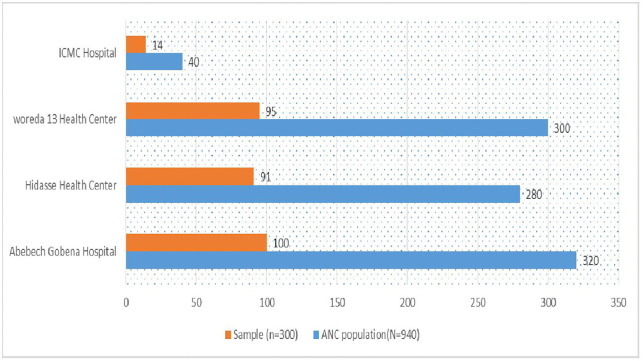
Proportional sample size allocation based on average monthly ANC attendance across study sites, Lemi Kura Subcity, Addis Ababa, Ethiopia, 2022.

The sample size was calculated by using unmatched case – control sample size calculation Epi Info ver. 7 application with 95% confidence level, 80% power, and key parameters from prior research indicating proportion of 32.1% versus 16.9% for women aged ≤24 among cases and controls respectively [20]. After adding a 10% allowance for non-response, a total of 300 participants (103 cases, 197 controls) were enrolled, with proportional allocated to selected sites as follows: Hidasse Health Center (n = 91), Woreda 13 Health Center (n = 95), Abebech Gobena Hospital (n = 100), and ICMC Hospital (n = 14) (Fig 2). Women who were critically ill or unable to participate were excluded. The timing of data collection and proportion of cases to controls were maintained in accordance with the allocated sample size.

**Study variables and Operational definition:**

- Dependent variable: Unintended pregnancy
- Independent variables: The variable that is manipulated or categorized to determine its impact on the dependent variable.
  - Socio-demographic factors: Age, marital status, educational level, religion, ethnicity, occupation, household income and residence.
  - Obstetric or reproductive factors such as Knowledge of FPM, the practice of FPM, Women’s autonomy, Parity and The ideal number of children.
  - Behavioral factors such as drinking alcohol, having multiple partners, smoking and khat chewing.

**Unintended pregnancy (UP):** is unwanted or mistimed pregnancy.

**Mistimed pregnancy:** is generally defined as a pregnancy that is desired later in life but not at conception, while some scholars defined as a pregnancy that occurred two or more years before a woman wanted to have a child. We used the general definition for this study.

**Unwanted pregnancy**: pregnancy that is not desired now, later or any time in the future describes.

**Autonomy**: woman’s ability to make and execute decisions regarding current pregnancy.

**Contraception**: is the act of intentionally preventing pregnancy, such as through the use of devices, practices, medications or surgical procedures.

**Short term contraceptive methods:** are contraceptive methods that can prevents pregnancy for not more than 3 months and requires frequent or regular user action. Such as male and female condoms, oral contraceptive pills, injectable and traditional methods.

**Long term contraceptive methods:** are contraceptive methods that can prevent pregnancy for more than one year without requiring frequent user action. Such as implant, IUCD, tubal ligation and vasectomy.

**Method failure:** the occupancy of unintended pregnancy while a woman is using a contraceptive method.

**The practice of family planning**: a woman who had used any contraceptive method previously.

**High Knowledge of family planning**: those study Participants who had answered correctly more than average knowledge questions regarding family planning methods.

**Low knowledge of family planning**: those study Participants who had answered average and below the average knowledge questions regarding family planning methods

**Alcohol use**: Any unit of home-brewed (e.g., Tela, Teje, Areka) as well as fabricated alcoholic beverages used during pregnancy.

**Khat use**: Any amount of khat used during pregnancy.

### Data collection tool and measurement

We collected data using a pretested, structured questionnaire adapted from validated instruments in published literature [33–38]. The questionnaire assessed: Socio-demographic characteristics, Obstetric and reproductive health history, Healthcare access and utilization, Behavioral factors and Pregnancy intention. For pregnancy intention, responses were classified in to “yes” or “no” categories to ensure clarity and consistency in case or control classification.

The questionnaire was developed initially in English, then professionally translated into Amharic and Afan Oromo by bilingual experts, followed by back-translated to ensure conceptual equivalence.

Data collection was conducted through face-to-face interviews at the selected health facilities. Eight professionally trained midwives served as data collectors, supervised by two field supervisors and the principal investigator. These personnel were neither students nor volunteers, they were selected based on their professional qualifications, prior experience in maternal health and ability to communicate effectively in local languages. Allocation to health facility was based on workload balance and facility size to ensure efficiency and coverage. To confirm pregnancy intention classification (not routinely documented in medical records), antenatal care providers used indirect questioning techniques, and responses were systematically coded. Instrument validity was further strengthened through pretesting on 5% of the sample at a non-study health facility, allowing refinement of wording, flow and coding procedures. During pretesting, sensitive questions related to abortion history were removed to improve participant comfort and data reliability.

### Data quality control

To ensure data quality, we implemented several strategies beyond questionnaire development and translation. Data entry was performed using a double entry system and discrepancies were cross checked to minimize error. No data were lost during data collection, incomplete responses were immediately refined with participants at the point of interview. The database was cleaned by checking for inconsistencies, outliers and missing values, with correction made through reference with original questionnaires. To reduce interviewer bias, data collectors received intensive training on standardized interviewing techniques, neutrality and confidentiality. Supervisor conducted daily reviews of completed questionnaire and provided feedback to ensure adherence to protocol. These measures collectively strengthened the reliability and validity of the dataset.

### Data processing and analysis

Data were coded, checked for completeness, and entered into Epi Info version 7, then exported to SPSS version 20 for analysis. Descriptive statistics were used to summarize the characteristics of cases and controls. Bivariate logistic regression was performed to assess the association between each independent variable and unintended pregnancy. Variables with a P-value < 0.25 were considered candidates for multivariable analysis.

A multivariable logistic regression model using the enter method was applied to identify independent predictors of unintended pregnancy by including all candidate variables simultaneously. Model fitness was assessed using the Hosmer-Lemeshow goodness-of-fit test (p > 0.05). The strength of association was reported using adjusted odds ratios (AOR) with 95% confidence intervals (CI), and statistical significance was declared set at p < 0.05.

### Ethics statement

The study was conducted according to the Helsinki Declaration for Human Subjects Research [38]. The Ethical approval was obtained from the Institutional Health Research Ethical Review Committee of the College of St. Paul Hospital Millennium Medical College (SPHMMC), Ethiopia (Ref.no: ሕ/ጤ/ት/716/2014 E.C). Written informed consent was secured from all participants after explaining the purpose and benefits of the study.

## Results

### Socio demographic characteristics of respondents

A total of 300 pregnant women (103 cases, 197 controls) attending antenatal care (ANC) were included in the study. All selected participants consented to and completed the interview, resulting in a 100% response rate. They ranged in age from 16 to 43 years (mean ± SD: 27.10 ± 4.73 years), with cases slightly older than controls (mean 27.61 vs 26.84 years, respectively). The majority (91% overall) were married (81.6% of cases and 85.9% of controls) and 89% cohabited with their partners, while the remainder (9% overall; 18.4% of cases and 4.0% of controls) were single, divorced, or widowed. The religious composition was predominantly Orthodox Christian (61.3% overall; 50.0% of cases and 67.0% of controls), with Muslims accounting for 20.3% and Protestants 18.4%. Ethnically, participants were mostly Amhara (45.3%) or Oromo (21.0%). In terms of residence, 62.2% lived in Lemi Kura sub-city of Addis Ababa, 23.0% in other sub-cities of Addis Ababa, and 14.0% in Oromia region. Regarding occupation, 36.0% were housewives, 27.0% were government employees, and 13.9% worked in the private or NGO sector. The mean monthly household income was 10,057.73 ± 7,130.57 birr (10,251 birr among cases vs 9,828.44 birr among controls). Educational attainment was evenly split, with 17% were illiterates and under primary, 28.3% were primary education and 54.6% were secondary and higher education completed. On average, participants lived 2.51 ± 1.62 km from the nearest health facility and 4.28 ± 3.17 km from the current ANC following site (Table 1).

**Table 1:** Socio-demographic characteristics of participants in Lemi kura Sub City, Addis Ababa, Ethiopia, 2022 (n = 300).

List of socio-demographic variables with category		control		case		Total		p-value
		n	%	n	%	N	%	
Age	16–24	55	27.90	27	26.20	82	27.30	0.13
	25–34	133	67.50	66	64.10	199	66.30	0.095
	35–43	9	4.60	10	9.70	19	6.30	
Marital status	single	4	2.00	13	12.60	17	5.70	0.370
	married	189	95.90	84	81.60	273	91.00	0.065
	widowed and divorce	4	2.00	6	5.80	10	3.30	0.530
The dwelling of the participant with partner	Same house	184	93.90	82	79.60	266	89.00	0.000
	Different place	12	6.10	21	20.40	33	11.00	0.122
Religion	Orthodox	132	67.00	52	50.50	184	61.30	0.023
	Muslim	34	17.30	26	25.40	60	20.30	0.887
	Protestant	31	15.80	25	23.30	55	18.40	0.424
Ethnicity	Amhara	97	49.20	39	37.90	136	45.30	0.059
	Oromo	36	18.30	27	26.20	63	21.00	0.939
	Gurage	16	8.10	6	5.80	22	7.30	0.219
	Tiger	11	5.60	4	3.90	15	5.00	0.274
	Others	37	18.80	27	26.20	64	21.30	0.213
Occupation of respondent	Housewife	74	37.90	36	35.00	110	36.90	0.869
	G. Employee	53	27.20	28	27.20	81	27.20	0.963
	Merchant	21	10.80	13	12.60	34	11.40	0.714
	Daily labor	20	10.30	12	11.70	32	10.70	0.767
	Other	27	13.90	14	5.80	41	13.40	0.046
Educational status	Illiterate	8	4.10	10	9.70	18	6.00	0.210
	read and write	21	10.70	12	11.70	33	11.00	0.775
	Primary	62	31.50	23	22.30	85	28.30	0.099
	Grade 9–12	58	29.40	27	26.20	85	28.30	0.318
	College	48	24.40	31	30.10	79	26.30	0.058
The average income of the household	< 3000	19	9.70	10	9.90	29	9.80	0.748
	3001 to 10,000	108	55.40	60	59.40	168	56.80	0.464
	>10,000	68	34.90	31	30.70	99	33.40	0.000
Distance from current ANC facility	< 4km	135	71.80	63	61.20	203	10.70	0.062
	> 4km	55	28.20	40	38.80	95	31.90	0.125

### Obstetric and reproductive characteristics of respondents

The majority of respondents in both groups reported having information about family planning methods, with 97.5% of controls and 96.1% of cases indicating awareness. Health workers were the primary source of information for 52.3% of controls and 58% of cases, followed by colleagues and others (26.7% of controls vs. 27% of cases), and media such as TV and radio (19% and 13%, control and case respectively).

Regarding beliefs, nearly all participants acknowledged the importance of family planning, with 97.5% of controls and 96.1% of cases expressing positive views. The most cited purpose of family planning was child spacing (52.1% of controls and 55% of cases), followed by benefits to maternal health, child health, and household economy.

Concerning knowledge of contraceptive types, 57.4% of controls and 44.7% of cases could identify methods suitable for both males and females. The average level of family planning knowledge was high for 76.6% of controls and 68% of cases. Ever use of family planning methods was similarly high in both groups (83.2% of controls and 83.5% of cases).

Among those who had used contraception, short-term methods were the most popular (59.1% of controls and 50% of cases), followed by long-term methods (37.8% of controls and 40.9% of cases), with natural methods being least utilized. The preference for contraceptive methods was primarily based on low side effects (24.4% of controls and 36% of cases, *p* = 0.02) and accessibility.

The majority accessed family planning services free of charge (53.1% of controls and 54.9% of cases), while about 39% of both groups found services reasonably priced.

Among the reasons for not using contraception prior to the current pregnancy, children’s desire was cited by 68.8% of controls and 28.6% of cases. Fear of side effects was reported by 12.5% of controls compared to 42.9% of cases. Other reasons like religious beliefs, inaccessibility, and partner domination were less frequently reported.

At the time of the survey, 53.3% of respondents were in their third trimester, with 56.1% of controls and 50% of cases falling in this category. Regarding antenatal care (ANC) initiation, half of the participants began ANC during the first trimester, with no significant difference between groups.

Incorrect utilization of contraceptives was the leading reason for the current pregnancy among cases (37.9%), followed by contraceptive failure (27.4%) and partner preference (15.8%). Among controls, the most cited reason was partner preference (85.7%).

Among cases reporting contraceptive failure, the most frequently failed methods were the calendar method (30.8%), followed by IUD (19.2%), injectable (15.4%), and post-pill (15.4%).

Regarding parity, 41.3% of controls and 35% of cases were nulliparous. Primiparous women made up 31.6% of controls and 20% of cases, while multiparous women accounted for 27% of controls and 45% of cases, showing a statistically significant difference (*p* = 0.02).

Ideal desired child number was three or more for 78.7% of controls and 58.3% of cases. Decision-making around pregnancy was predominantly joint in both groups, with 77.2% of controls and 60.1% of cases deciding together with their partners. However, decision-making by the partner alone was more common among cases (29.1%) than controls (11.7%, *p* = 0.04) (Table 2).

**Table 2:** Obstetric and reproductive health related characteristics of unintended pregnancy, Lemi Kura sub-city, Addis Ababa, Ethiopia, 2022 (n = 300).

variables	Categories	Controls %	Cases %	Total %	p- value
having information about family planning	Have	192 (97.5)	99 (96.1)	291 (97)	0.520
	Don’t have	5 (2.5)	4 (3.9)	9 (3)	0.739
sources of information about family planning methods	printed materials	3 (1.5)	2 (2)	5 (1.7)	1.000
	TV and Radio	37 (19)	13 (13)	50 (16.9)	1.000
	Health workers	102(52.3)	58 (58)	160 (54.2)	1.000
	colleagues& others	53(26.7)	27 (27)	80 (26.8)	1.000
believe in importance of family planning	Yes	192 (97.5)	98 (96.1)	290 (97)	0.510
	No	5 (2.5)	4 (3.6)	9 (3)	0.739
importance of family planning	child Spacing	100 (52.1)	55 (55)	155 (53.1)	0.286
	Maternal health	36 (18.8)	12 (12)	48 (16.4)	0.081
	child Health	12 (6.2)	9 (9)	21 (7.2)	0.678
	economy	44 (21.9)	24 (24)	54 (23.3)	0.202
know types FPM for males and females	Have	113 (57.4)	46 (44.7)	159 (53)	0.001
	Don’t have	84 (42.6)	57 (55.3)	141 (47)	0.156
average family planning know	high	151(76.6)	70 (68)	221 (73.7)	0.106
	poor	46 (23.4)	33 (32)	79 (26.3)	0.145
Ever used FPM	Yes	164 (83.2)	86 (83.5)	250 (83.3)	0.957
	No	33 (16.8)	17 (16.5)	50 (16.7)	0.026
Type of family planning method used	Natural	5 (3)	8 (9.1)	13 (5.2)	0.095
	Short term	97 (59.1)	44 (50)	141 (56)	0.373
	Long term	62 (37.8)	36 (40.9)	98 (38.9)	0.009
Reason for preferred method	low side effect	39 (24.4)	31 (36)	70 (28.5)	0.023
	accessibility	37 (23.1)	25 (29.1)	62 (25.2)	0.074
	high acceptance	35 (21.9)	15 (17.4)	50 (20.3)	0.558
	low cost	4 (2.5)	0 (0)	4 (1.6)	0.999
	duration	45 (28.1)	15 (17.4)	60 (24.4)	0.000
cost of family planning methods	free of charge	104 (53.1)	56 (54.9)	160 (53.7)	0.023
	reasonably priced	77 (39.3)	39 (38.2)	116 (38.9)	0.074
	moderately expe	12 (6.1)	6 (5.9)	18 (6)	0.558
	very expensive	3 (1.5)	1 (1)	4 (1.3)	0.999
reason for not using Family planning	information Lack	0(0)	1(7.1)	1(2.2)	1.000
	children desire	22(68.8)	4(28.6)	26(56.5)	1.000
	Religious view	6(18.8)	1(7.1)	7(15.2)	1.000
	Fear of side effects	4(12.5)	6(42.9)	10(21.7)	1.000
	Inaccessibility	0(0)	1(7.1)	1(2.2)	1.000
	Partner domination	0(0)	1(7.1)	1(2.2)	1.000
the current stage of pregnancy	1st trimester	15(7.7)	12(11.7)	27(9)	0.182
	2nd trimester	71(36.2)	41(39.8)	112(37.5)	0.357
	3rd trimester	110(56.1)	50(50)	160(53.3)	0.000
ANC started period this pregnancy	1st trimester	101(51.5)	50(48.5)	151(50.5)	0.991
	2nd trimester	91(46.4)	51(49.5)	142(47.5)	0.897
	3rd trimester	4(2)	2(1.9)	6(2)	0.423
reason for current pregnancy	unavailability	1(14.3)	16(16.8)	17(16.7)	0.999
	Incorrect utilization	0(0)	36(37.9)	36(35.3)	1.000
	Failure of coc used	0(0)	26(27.4)	26(25.5)	1.000
	Partner preference	6(85.7)	15(15.8)	21(20.6)	0.999
	Rape	0(0)	2(2.1)	2(2)	0.999
failed contraceptive for this pregnancy	Calendar		8(30.8)		
	Condoms		1(3.8)		
	Pills		3(11.5)		
	Injectable		4(15.4)		
	post-pill		4(15.4)		
	Implanon		1(3.8)		
	IUD		5(19.2)		
Parity	nulliparous	81(41.3)	35(35)	116(39.2)	0.018
	primiparous	62(31.6)	20(20)	82(27.7)	0.003
	multiparous	53(27)	45(45)	98(33.1)	0.420
ideal child number	< 2	42(21.3)	43(41.7)	85(28.3)	0.000
	>3	155(78.7)	60(58.3)	215(71.7)	0.000
decision-maker of pregnancy	By themselves	22(11.2)	11(10.8)	22(7.4)	0.610
	By their partner	23(11.7)	30(29.1)	33(11.7)	0.000
	Both of them	152(77.2)	62(60.1)	242(80.9)	0.000

**Fig 3:**
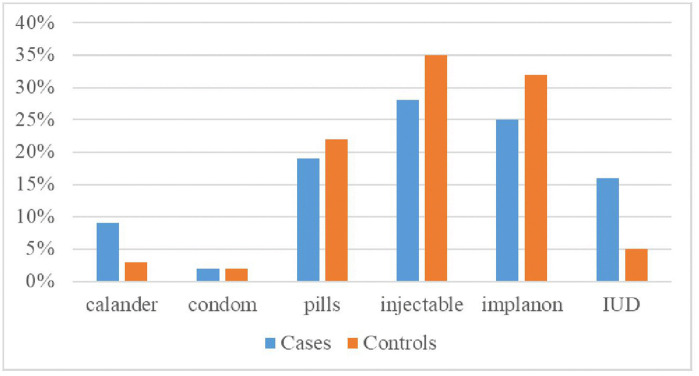
Types of family planning methods practiced among study participants Lemi Kura Sub city, Addis Ababa, Ethiopia. 2022 (n = 300).

Out of 250 (83.3%) participant practiced family planning method those used calendar were 5.2% (9.1% case and 3% control), condoms were 2% (2.3% case and 1.8% control), pills were 21% (19.3% case and 22% control), injectable were 32.9% (28.4% case and 35.4% control), Implanon were 29.8% (25% case and 32.3% control) and IUCD were 9.1% (15.9% case and 5.5%control) (Fig 3).

### Behavioral characteristics of study participants

In this study, 10.7% of the participants reported drinking alcohol, with a higher proportion among cases (15.5%) compared to controls (8.2%). Khat chewing and cigarette smoking were each reported by 0.7% of participants, both slightly higher among cases (2.0%) than controls (1.0% and 0.5%, respectively). Additionally, having multiple sexual partners was more common among cases (23.3%) than controls (4.1%) (Table 3).

**Table 3:** Behavioral characteristics of unintended pregnancy, Lemi Kura sub-city, Addis Ababa, = Ethiopia, 2022 (n = 300).

behavioral characteristics	Category	Frequency(%)			P-value
**Controls (n = 197)**	**Cases (n = 103)**	**Total**
Alcohol	Drinking	16(8.20)	16(15.50)	32(10.70)	0.06
	Not drinking	179(91.80)	87(84.50)	266(89.30)	0.00
khate	chewing	2(1.00)	0(0)	2(0.70)	
	Not chewing	195(99)	102(100)	297(99.30)	
smoking	smoker	1(0.50)	1(1.00)	2(0.70)	
	Not smoker	196(99.50)	101(99)	297(99.30)	
Multiple sexual partners	Have	8(4.10)	24(23.30)	32(10.70)	0.00
	Don’t have	189(95.90)	79(76.70)	268(89.30)	0.00

### Factors affecting an unintended pregnancy

Accordingly, out of 16 variables that met the bivariate selection criterion and were entered in to the multivariable model, 7 variables remain statistically significant and were identified as determinants of unintended pregnancy after adjustment for potential confounders.

Variables identified as candidate determinants of unintended pregnancy during bivariate logistic regression analysis were age of woman, marital status, dwelling status, religion, ethnicity, educational status, distance from an ANC service (a health facility), average knowledge about Family planning method, ever use of any type of family planning method, types of Family planning method they had used, reason for family planning method preference, parity, ideal number of children they need to have, the process how decision made about current pregnancy, experienced alcohol drinking and having multiple sexual partners (see Table 4).

**Table 4:** Adjustment variables included in the final multivariable logistic regression model across sociodemographic, obstetric and reproductive, and behavioral domains were.

Domain	Variable
**Sociodemographic**	• Age • marital status • dwelling status • religion • ethnicity and • educational status • monthly income
**Obstetric and Reproductive factors**	• distance from an ANC service (a health facility) • knowledge about Family planning method, • practice of family planning method • types of Family planning method they had used • parity • ideal number of children they need to have • women autonomy
**Behavioral factors**	• experienced alcohol drinking • having multiple sexual partners

However, on multivariable logistic regression analysis, identified variables as independent predictors of unintended pregnancy were religion, distance from an ANC service (a health facility), and reason for family planning method preference, decision maker of pregnancy, parity, ideal child number, and having multiple sexual partners (see Table 4).

Muslim and Protestant women had 3.33 and 3.06 times the odds of experiencing an unintended pregnancy, respectively, compared to Orthodox Christian women (Muslim: AOR = 3.33; 95% CI: 1.21–9.15; p < 0.02; Protestant: AOR = 3.06; 95% CI: 1.07–8.75; p < 0.04).

Women who traveled 4 km or more from their home to the health facility for current ANC services had 2.70 times the odds of experiencing an unintended pregnancy compared to those who traveled less than 4 km (AOR = 2.70; 95% CI: 1.07–8.75; p < 0.02).

Compared to women who cited duration as their reason for preferring a family planning method, those who cited accessibility had 5.92 times the odds of experiencing an unintended pregnancy (AOR = 5.92; 95% CI: 1.41–24.79; p < 0.02).

Multiparous women had 5.37 times the odds of experiencing an unintended pregnancy compared to nulliparous women (AOR = 5.37; 95% CI: 1.60–18.15; p < 0.01).

Women whose ideal number of children was less than or equal to two had 3.63 times the odds of experiencing an unintended pregnancy compared to women whose ideal number of children was three or more (AOR = 3.63; 95% CI: 1.50–9.01; p < 0.01).

Women whose current pregnancy decision was made by their partner had 16.58 times the odds of experiencing an unintended pregnancy compared to those who made the decision themselves (AOR = 16.58; 95% CI: 2.40–114.15; p < 0.01).

Women with multiple sexual partners had 6.46 times the odds of experiencing an unintended pregnancy compared to women with a single partner (AOR = 6.46; 95% CI: 1.38–30.38; p < 0.02) (Table 5).

**Table 5:** Factors associated with unintended pregnancy among pregnant women attending the ANC services at selected health care facilities, Lami Kura subcity, Addis Ababa, 2022.

Variable	Category	Controls	Cases	COR(95% CI)	AOR(95% CI)	P-Value
Religion	Orthodox	132	52	0.39		1
	Muslim	34	26	1.941(1.06,3.55)	3.33(1.21,9.15)	0.02
	Protestant	31	25	2.047(1.11,3.79)	3.06(1.07,8.75)	0.04
Distance from ANC service	<4KM	135	63	1.62(0.98,2.68)		
	>=4KM	55	40	0.45	2.70(1.07,8.75)	0.02
Reason for FPM preference	low side effect	39	31	2.39(1.13,5.053)	2.92(1,8.6)	0.05
	Accessibility	37	25	2.03(0.94,4.40)	5.92(1.41,24.79)	0.02
	High acceptance	35	15	1.29(0.55,2.98)	2.56(0.66,9.89)	0.17
	Duration	45	15	0.33		
Decision maker of pregnancy	by themselves	22	11			
	by their partner	23	30	2.61(1.06,6.45)	16.58(2.4,114.15)	0.01
	both of them	152	62	0.82(0.37,1.78)	1.94(0.35,10.72)	0.45
Parity	Nulliparous	81	35	0.43		
	Primiparous	62	20	0.75(0.39,1.42)	2.01(0.60,6.76)	0.26
	Multiparous	53	45	1.97(1.12,3.44)	5.37(1.60,18.15)	0.01
Ideal child number	<=2	42	43	2.65(1.57,4.45)	3.63(1.50,9.01)	0.01
	>=3	155	60	0.39		
Having multiple sexual partners	yes	8	24	7.37(3.03,17.94)	6.46(1.38,30.38)	0.02
	no	189	79	0.426		

## Discussion

### Socio demographic factors

The results of this study indicated that unintended pregnancy could occur as the result of socio-demographic factors like being Protestant or Muslim instead of Orthodox and visiting the health facilities somewhat far at least more than or equal to 4 km from their residence for ANC follow up.

Muslim and Protestant pregnant women had 3.33 and 3.06 times higher odds of unintended pregnancy compared to Orthodox women, consistent with studies in India [21] (Muslim) and Wolayita, Ethiopia [26] (Protestant). This disparity may stem from lower autonomy and pronatalism in Muslim communities [21] and Protestant religious teachings discouraging contraceptive use [26]. The observed differences from Ethiopian findings [39] could reflect variations in study design. Pregnant women who traveled longer distances to ANC facilities had 2.70 times higher odds of unintended pregnancy compared to those visiting nearby facilities, aligning with studies in Ethiopia [24] and Kersa, eastern Ethiopia [7]. This disparity may reflect lower service quality at local facilities and insufficient integration of family planning outreach programs with the Oromia Health Bureau in outlying areas like Finfine Zuria.

### Obstetric and reproductive factors

The results of this study indicated that unintended pregnancy could occur as the result of obstetric factors like dependent women instead of self-autonomous, being multiparous instead of nulli/ primiparous and when the Ideal number of children decreases.

Multiparous women had 5.37 times higher odds of unintended pregnancy compared to nulliparous women, consistent with findings from Ecuador [19], Bangladesh [16], and Ethiopia [24]. This likely reflects lower fertility desire among multiparous women due to accumulated gynecological, obstetric, and economic burdens.

Women desiring ≤2 children had 3.63 times higher odds of unintended pregnancy compared to those wanting ≥3 children, indicating an inverse relationship between ideal family size and unintended pregnancy. This aligns with findings from UNFPA [1], Nepal [9], and Ganji-Oromia, Ethiopia [3], potentially reflecting women’s limited autonomy in reproductive decision-making.

Women with low autonomy had 16.58 times higher odds of unintended pregnancy compared to autonomous women, consistent with findings from UNFPA [1], Bangladesh [16], Tanzania [31], and Ethiopia [37]. This suggests enhancing women’s autonomy could address gender inequities while reducing unintended pregnancy and improving maternal-child health outcomes. The contrasting results from Nepal [9] may reflect methodological differences in autonomy measurement.

### Behavioral factors

The results of this study indicated that unintended pregnancy could occur as the result of behavioral factor like having multiple sexual partners.

Women with multiple sexual partners had 6.46 times higher odds of unintended pregnancy than those with one partner, aligning with studies in China [33] and Tanzania [35]. Potential explanations include inconsistent contraceptive use, alcohol-related risk behaviors, and reduced partner accountability in multi-partner relationships.

## Conclusions

This study identified several important predictors of unintended pregnancy, including partner-led pregnancy decisions, having multiple sexual partners, multiparty, a lower ideal number of children, religious affiliation, greater distance to health facilities for ANC services, and reasons for family planning method preference. These findings highlight the need for targeted interventions that promote women’s autonomy in reproductive decision-making, improve access to comprehensive family planning services, and address sociocultural and service accessibility barriers to reduce unintended pregnancies. Therefore, in family planning programs, those women having two or more children and multiple sexual partners should be the focus of special attention by assuring the accessibility of contraceptives and creating clear awareness of the side effects of the programs to prevent unintended pregnancy and its consequences.

### Strengths and limitations of study

As pregnancy advances to term or after delivery, women often reinterpret unintended pregnancies as “happy accidents.” During data collection, unintended pregnancies were more common in the 1st and 2nd trimesters, while women in the 3rd trimester tended to remain silent, with many later viewing their pregnancies positively. This study incorporated multiple determinants of unintended pregnancy while omitting sensitive questions such as abortion history. Selection bias was minimized by recruiting both cases and controls from the same health facilities using consecutive sampling. Recall bias was limited since most questions focused on basic healthcare and personal opinions, though variables like ANC history may have been affected. Women with unintended pregnancies may avoid ANC follow-up, potentially introducing selection bias. Finally, the findings may not be generalizable to women who do not attend health facilities

## Supporting information

S1 Data Dataset_deidentified. (SAV)
