# Supplementary material for: Determinants of an unintended pregnancy among women attending ante natal care at health facilities in Lemi Kura sub city, Addis Ababa, Ethiopia: Institution based case-control study
Source: PLOS Glob Public Health. 2026 Jul 9;6(7):e0004920. doi: 10.1371/journal.pgph.0004920 (PMC13349197; doi:10.1371/journal.pgph.0004920)
Supplement: Attachment — Submitted filename: Rev_1.pdf [file pgph.0004920.s002.pdf]

Determinants of an Unintended Pregnancy among Women Attending Ante Natal Care at Health Facilities in Lemi Kura Sub City, Addis Ababa, Ethiopia: Institution based Case-Control Study

INTRODUCTION

(I-L1) Unintended pregnancy (UP) is a significant public health concern worldwide. The text can be improved to say that unintended pregnancy (UP) is a public health concern. There is no insignificant public health problem.

The bridge between the first and second paragraphs needs improvement. In the first paragraph, the writer should have opened the question of the nature of the consequences of unintended pregnancy. And in the second paragraph, these consequences would be detailed. Do note that in the third paragraph, the author discusses the causes of unintended pregnancy, but the consequences of the study phenomenon reappear in the fourth paragraph. The text does not flow naturally.

(I-L7-10) The first citation from the third literature source has about 43 words. Does the journal's guideline have a specific approach for lengthy quotations?

The text sequence: (I-L13-4) burden of unintended pregnancy is disproportionately higher in developing countries compared to developed nations. (I-L7-8) And unintended pregnancies contribute substantially to maternal morbidity and mortality, particularly in low-resource settings where access to maternal healthcare services. They can stay together.

(I-L14-5) In 2010, an estimated 80 million unintended pregnancies occurred globally, with approximately 75 million occurring in low-income regions. This statistic is from more than a decade ago. Do you have any data from the last 5 years?

(I-L15-7) Despite international efforts to address the issue, unintended pregnancy rates remain high, particularly in sub-Saharan Africa. What is the value in the sub-Saharan Africa region, and what is the reflection behind it?

(I-L-21) A study conducted by the Guttmacher Institute in Ethiopia reported that 101 unintended pregnancies occurred per 1,000 women aged 15-44, with 42% of all pregnancies classified as unintended. Can you make it more clear, please?

(I-L-26) Globally, unintended pregnancies account for a substantial proportion of unsafe abortions. Of the estimated 210 million pregnancies occurring annually, approximately 38% (how many?) are unintended. Out of those X unintended pregnancies, 22% result in abortion. Do you mean it?

In the sixth paragraph, the central topic is unclear. You can combine the information on the determinants with the information on the socio-demographic factors described in the third paragraph.

The penultimate one and the last paragraphs can be combined into a single paragraph.

General Comment: The text contains fundamental information for the purpose of the study. However, it needs to update the information, provide context, and improve the writing and structuring of the paragraphs in order to ensure greater textual fluency and comprehension by the readers.

METHODS AND MATERIALS

Study design and setting

Why did you select hospitals and centers knowing that they are institutions of different levels?

Do patients who attend the health centers have the same sociodemographic characteristics as those who attend the Hospitals?

To provide a better understanding of the context, you can include information about family planning, local statistics on unwanted pregnancies, and abortion laws.

Population and sampling

We conducted an unmatched case-control. This text shoul included in previous section.

The text can be improved to say that the study population were pregnant women attending antenatal care (ANC) in Health Facilities in Lemi Kura.

Which random selection technique was used to select the health units? How did you calculate to select 4 health units out of the 14 that offer the antenatal services?

By proportionally allocated across sites, do you mean it is proportionally sampled? If yes, how did you do it?

From the abstract, this is a cluster random sampling technique. Please, how did you select the sample within the clusters?

Note that 197 controls are not double the 103 cases. Did you lose any data?

Please specify whether the study included only women residing in that area or all. Was there an age cutoff for selecting participants? If yes, what was it based on?

Data collection tool and measurement

I think that pregnancy intention should be classifed as yes or not.

Well trained personnel conducted face-to-face interviews at the selected health facilities. Who they are (student or volunters)?

Did you use any criaterial to allocate the personnel in the health units?

Did you use any statistical tools to validate the instruments?

Data Quality Control

This section has only repeated information, therefore, I think it is umnecessary.
Did you lose any data?
How did you clean the database?
Did you use any strategy to minimize the interviewer bias?

Data Processing and Analysis

I believe you used an enter model and not backward stepwise (likelihood ratio) to determine predictors of unintended pregnancy. The marginal α=0.25 may be less conservative than 0.15, 0.10, and 0.05.

Operational definition

It sould be included after population and sampling section and called as study variable and operational definition. Please, let we know about the depending and independing variable and their definition.

General Comment: Regarding methodology, a profound change is needed in order to provide information on the key aspects of the paths to achieving the objectives. There are no details of key elements for validating the study. And also, the authors do not refer any ethical implication of their study.

RESULTS

Socio demographic characteristics of Respondents

Why claim a 100% response rate if the number of control cases is not double the number of cases?

When you compare case with controls, make sense to use a significance test for that. It is the Table, but you did not report in the text. Which association test did you use to compare the cases and controls at the beginning? This is not reported in the data analysis section.

The study aimed to identify the determinants of unintended pregnancy among women attending antenatal care services in health facilities within Lemi Kura sub-city, Addis Ababa, Ethiopia. Based on the literature, what is the relationship between unwanted pregnancy and the distance from home to antenatal care services?

I advise you to separate de literacy status and the education status. At the same time, they do not have exclusive categories.

Obstetric and Reproductive Characteristics of Respondents

Why not include the significance test in Table 2, as you did in Table 1 and 3?
It would be fine to explore the history of abortion to make sense of what was reported in the introduction.

Believe in importance of family planning? I think this type of question would be assessed with an appropriate instrument, not by simply self-report.

Behavioral characteristics of study participants

It is unclear why the p-values for the smoking habit category variables are missing. These variables could be grouped into a single variable to improve comparability across strata.

Factors affecting the unintended pregnancy

In the Bivariate logistic regression was not included all study variables. What criteria and tests did you apply to select the eligible variable? Please, write it in the data analysis section. Is there any sense to include the p-value and confidence intervals in the same table?

DISCUSSION

The discussion should be separated into sections according to determinant´s nature.

A mistake in selecting the study variables for analysis can lead to uncertain discussion and conclusion. In a case-control study, the focus is on prior exposures that influenced the outcome. Therefore, distance to antenatal services is not relevant to this analysis.

The authors did not refer any study limitations and suggestions for future research direction.

Thank you...
