# Supplementary material for: Determinants of an unintended pregnancy among women attending ante natal care at health facilities in Lemi Kura sub city, Addis Ababa, Ethiopia: Institution based case-control study
Source: PLOS Glob Public Health. 2026 Jul 9;6(7):e0004920. doi: 10.1371/journal.pgph.0004920 (PMC13349197; doi:10.1371/journal.pgph.0004920)
Supplement: Attachment — Submitted filename: Response to Reviewers.pdf [file pgph.0004920.s005.pdf]

INTRODUCTION

**Reviewer Comment**	**Authors' Agreement and Rebuttal Response**
(I-L1) Unintended pregnancy (UP) is a significant public health concern worldwide. The text can be improved to say that unintended pregnancy (UP) is a public health concern. There is no insignificant public health problem.	While we understand the reviewer's concern, the term "significant public health concern" is widely used in scientific literature to indicate the magnitude and impact of a specific issue. The phrasing does not imply that other public health problems are insignificant but helps clarify the substantial burden associated with unintended pregnancy. We therefore prefer to retain the original wording for accuracy and alignment with standard scholarly practice.
The bridge between the first and second paragraphs needs improvement. In the first paragraph, the writer should have opened the question of the nature of the consequences of unintended pregnancy. And in the second paragraph, these consequences would be detailed. Do note that in the third paragraph, the author discusses the causes of unintended pregnancy, but the consequences of the study phenomenon reappear in the fourth paragraph. The text does not flow naturally.	We appreciate the reviewer's observation regarding the flow between paragraphs. The comment correctly highlights that the transition from the first to the second paragraph can be strengthened by more explicitly introducing the consequences of unintended pregnancy (UP) at the end of the first paragraph. Additionally, the reviewer is correct that the discussion of consequences appears again in the fourth paragraph, which may disrupt the overall narrative structure. We agree that reorganizing the placement of causes and consequences could enhance coherence and we addressed it through track change. While we acknowledge the reviewer's concern, we would like to clarify that the current structure was intentionally designed to first define UP and briefly mention its broad implications in the opening paragraph. The second paragraph then expands on the health-related consequences in greater

	detail, aligning with standard scientific writing practices that move from general definitions to specific impacts. The fourth paragraph addresses a *specific* consequence—unsafe abortion—which is treated separately due to its major public health significance. This separation aims to provide emphasis rather than disrupt flow.
(I-L7-10) The first citation from the third literature source has about 43 words. Does the journal's guideline have a specific approach for lengthy quotations?	Thank you for noting the length of the quotation. We understand that extended excerpts should be used sparingly. In accordance with the journal's preferences, we have replaced the 43-word quotation with a concise paraphrase while retaining the original meaning.
The text sequence: (I-L13-4) burden of unintended pregnancy is disproportionately higher in developing countries compared to developed nations. (I-L7-8) And unintended pregnancies contribute substantially to maternal morbidity and mortality, particularly in low-resource settings where access to maternal healthcare services. They can stay together.	Thank you for the comment. These two statements were intentionally presented together because lines I-L7–8 address the gynecological effects of unintended pregnancy, while lines I-L13–14 discuss its prevalence in populations with limited access to maternal health services. Therefore, we believe it is appropriate to keep the two sentences as they are.
(I-L14-5) In 2010, an estimated 80 million unintended pregnancies occurred globally, with approximately 75 million occurring in low-income regions. This statistic is from more than a decade ago. Do you have any data from the last 5 years?	Thank you for this important observation. We agree that the statistic from 2010 is outdated. We have now replaced it with more recent global estimates published within the past five years to ensure the introduction reflects current evidence.

In the sixth paragraph, the central topic is unclear. You can combine the information on the determinants with the information on the socio-demographic factors described in the third paragraph.	We agree that the paragraph would be well organized and we did it through track change because, discussion of determinants such as parity, age at marriage, media exposure, and religious beliefs overlaps with the socio-demographic factors already mentioned earlier, including socioeconomic status, contraceptive access, reproductive age, and educational level. Combining these related elements into a single, coherent section would strengthen the logical flow and prevent fragmentation.
(I-L15-7) Despite international efforts to address the issue, unintended pregnancy rates remain high, particularly in sub-Saharan Africa. What is the value in the sub-Saharan Africa region, and what is the reflection behind it?	This reflects persistent structural barriers—such as limited contraceptive access, inadequate health-system capacity, socioeconomic inequality, and restrictive gender norms—that continue to hinder progress. International efforts alone are insufficient unless paired with long-term local investments, culturally grounded strategies, and expanded reproductive-health autonomy for women and adolescents. So that we have briefly discussed this reason in the manuscript.
(I-L-21) A study conducted by the Guttmacher Institute in Ethiopia reported that 101 unintended pregnancies occurred per 1,000 women aged 15-44, with 42% of all pregnancies classified as unintended. Can you make it more clear, please?	A study in Ethiopia found that for every 1,000 women aged 15–44, about 101 experienced an unintended pregnancy in a given year. This means unintended pregnancies are relatively common. The study also showed that 42% of all pregnancies in the country were not planned—either unwanted at the time or mistimed

(I-L-26) Globally, unintended pregnancies account for a substantial proportion of unsafe abortions. Of the estimated 210 million pregnancies occurring annually, approximately 38% (how many?) are unintended. Out of those X unintended pregnancies, 22% result in abortion. Do you mean it?	We have understood the Numbers from the literature<br><br>38% of 210 million pregnancies → ≈ 79.8 million unintended pregnancies<br><br>22% of these unintended pregnancies end in abortion → ≈ 17.6 million abortions<br><br>So yes, the statement means almost 80 million unintended pregnancies, and about 18 million abortions each year globally.
In the sixth paragraph, the central topic is unclear. You can combine the information on the determinants with the information on the socio-demographic factors described in the third paragraph.	Thank you for your excellent suggestion even though we were tried to describe general and local level separately, combining these related elements into a single, coherent section would strengthen the logical flow and prevent fragmentation. So that we did it as your suggestion.
The penultimate one and the last paragraphs can be combined into a single paragraph.	Thank you for the excellent suggestion. We agree that combining the penultimate and final paragraphs will create a more cohesive and impactful conclusion. So that we implemented that change accordingly.
The text contains fundamental information for the purpose of the study. However, it needs to update the information, provide context, and improve the writing and structuring of the paragraphs in order to ensure greater textual fluency and comprehension by	Thank you for your thorough and constructive feedback. we agree that the text serves to lay the fundamental information for the study's purpose.<br><br>Regarding your points on updating the information and providing more context, we would like to clarify our intention. The information in this section is deliberately foundational, establishing the historical baseline from which the

the readers.	study's novel contributions and updated data are introduced in subsequent chapters. The broader context is intentionally built up progressively to support the study's central argument. However, we wholeheartedly agree with your assessment that the writing and paragraph structure need improvement. So that the revision done to strengthen the manuscript's overall impact.
Authors have largely used old references	Thank you for highlighting this. We agree that incorporating more recent studies will strengthen the review. So that we have updated the literature reviewed, these additions provide a more current and comprehensive view of the field and better contextualize the study's contribution.

2. Methodology

**Reviewer Comment**	**Authors' Agreement and Rebuttal Response**
Operational definition It should be included after population and sampling section and called as study variable and operational definition. Please, let we know about the depending and independing variable and their definition. "Operational definitions should cover the definitions of all the important variables such as unintended pregnancy, short term and long term contraceptive methods, knowledge of the	Your point is correct, so that we addressed operational definition section after the Population and Sampling section and is typically titled "Study Variables and Operational Definitions." Including this section helps readers understand exactly how each variable in the study is defined, measured, and categorized. It ensures clarity, consistency, and reproducibility

methods, average knowledge of the methods, method failure etc”	
Study design and setting Why did you select hospitals and centers knowing that they are institutions of different levels? Do patients who attend the health centers have the same sociodemographic characteristics as those who attend the Hospitals? To provide a better understanding of the context, you can include information about family planning, local statistics on unwanted pregnancies, and abortion laws.	Thank you for this thoughtful comment. We intentionally included both hospitals and health centers as well as private and government health facilities to ensure that the sample reflected the full understanding on determinants of unintended pregnancy in Lami kura subcity. Women seek services at different facility levels based on factors such as proximity, perceived quality of care and availability of specific services. Including multiple levels allowed us to capture a more representative range of client experiences and minimized selection bias toward any single type of facility. Since our study populations were those of attending antenatal follow up we were not concerned on clients of receiving family planning and abortion care services. For your information in all health centers and hospitals in Ethiopia there are abortion care service and family planning service provision units.
Population and sampling We conducted an unmatched case-control. This text shoul included in previous section. The text can be improved to say that the study population were pregnant women attending antenatal care (ANC) in Health Facilities in Lemi Kura. Which random selection technique	Thank you for the insightful comments that helped strengthen the clarity and methodological transparency of the study. We have revised the section accordingly.<br>1. Clarification of the Study Population<br>The revised text now states that the study population consisted of pregnant

was used to select the health units? How did you calculate to select 4 health units out of the 14 that offer the antenatal services? By proportionally allocated across sites, do you mean it is proportionally sampled? If yes, how did you do it? From the abstract, this is a cluster random sampling technique. Please, how did you select the sample within the clusters? Note that 197 controls are not double the 103 cases. Did you lose any data? Please specify whether the study included only women residing in that area or all. Was there an age cutoff for selecting participants? If yes, what was it based on?	women attending antenatal care services in health facilities located in Lemi Kura sub-city.<br><br>2. Selection of Health Facilities<br><br>We agree that the sampling technique used to choose participating health facilities needed further explanation. We have clarified that a cluster random sampling approach was applied. From the 14 health facilities offering antenatal care, four were selected using a simple random technique (lottery method). The rationale was to obtain a representative sample while maintaining feasibility.<br><br>3. Proportional Allocation Across Sites<br><br>We confirm that proportional sampling was used. The number of participants drawn from each selected health facility was calculated based on the proportion of pregnant women attending ANC in that facility relative to the total ANC attendance across all selected sites. Within each selected facility, systematic random sampling was employed. Sampling intervals were calculated using the flow of ANC attendees during the data-collection period.<br><br>4. Ratio of Cases to Controls<br><br>You are correct that the number of controls is not exactly double the number of cases. No data were lost. The final numbers reflect the actual availability of eligible cases during the study period, while all selected controls were retained.

	5. Inclusion Based on Residence<br><br>We agree that this needed specification. The study included pregnant women who either resided in the Lemi Kura area or were receiving ANC services there, regardless of residence.<br><br>6. Age Cutoff<br><br>All pregnant women of reproductive age (15–49 years) were eligible, consistent with standard maternal health research practices.
Sample size calculation is not clear or not clearly written	Thank you for your comment, so that we refined it to be cleared enough for readers though the sample size calculation was originally correct.
Data collection tool and measurement I think that pregnancy intention should be classifed as yes or not. Well trained personnel conducted face-to-face interviews at the selected health facilities. Who they are (student or volunters)? Did you use any criaterial to allocate the personnel in the health units? Did you use any statistical tools to validate the instruments?	To address this, we have specified that trained midwives—not students or volunteers—conducted the interviews, and allocation was based on professional criteria and facility workload. While the reviewer suggested further statistical validation of the instrument, we respectfully note that our questionnaire was adapted from previously validated instruments in published literature. To ensure contextual appropriateness, we conducted pretesting on 5% of the sample, which is a standard methodological practice for instrument validation in field studies. This process allowed us to refine the tool and confirm its reliability in the local setting. Thus, while additional statistical validation (e.g., factor analysis) could be

	considered, the combination of adaptation from validated sources and pretesting provided sufficient rigor for this study.
Data Quality Control This section has only repeated information, therefore, I think it is umnecessary. Did you lose any data? How did you clean the database? Did you use any strategy to minimize the interviewer bias?	We agree with the reviewer that the initial version of the Data Quality Control section contained some repetition of information already described under data collection. We have therefore refined the section to focus on data management, cleaning procedures, and strategies to minimize interviewer bias, which were not previously detailed. While the reviewer suggested that the section was unnecessary, we respectfully note that data quality control is a critical methodological component that ensures the credibility of study findings. By explicitly documenting how data were cleaned, how missing values were handled, and how interviewer bias was minimized, we provide transparency and strengthen the rigor of our study. Thus, we believe the section is essential, though we have revised it to avoid redundancy and highlight unique quality assurance measures.

Data Processing and Analysis I believe you used an enter model and not backward stepwise (likelihood ratio) to determine predictors of unintended pregnancy. The marginal α=0.25 may be less conservative than 0.15, 0.10, and 0.05.	We agree with the reviewer that our initial description of the regression method was imprecise. The analysis was conducted using an enter model approach, not backward stepwise likelihood ratio, and we have revised the section accordingly. We also acknowledge the reviewer's point that a marginal α = 0.25 may be less conservative compared to thresholds such as 0.15 or 0.10. Our choice of 0.25 was based on established methodological recommendations to avoid excluding potentially important variables at the bivariate stage, but we recognize the merit of more conservative cutoffs and have clarified our rationale. While the reviewer suggests that α = 0.25 may be less conservative, we respectfully note that this threshold is widely used in epidemiological studies to ensure that variables with potential confounding effects are not prematurely excluded from multivariable models. Using a more conservative threshold (e.g., 0.10 or 0.15) could risk omitting variables that may become significant after adjustment. Thus, our choice of 0.25 was intentional and consistent with best practices in logistic regression modeling. Nevertheless, we have clarified the rationale in the revised text to avoid ambiguity.
The authors do not refer any ethical implication of their study.	Thank you for your valuable feedback. We have made substantial revisions to the methodology section to clearly demonstrate how the study achieved its objectives. In the previous version of the manuscript, the ethics statement was placed after the discussion section; however, we have now moved it to the

	methodology section to ensure it is more visible and appropriately positioned.
Regarding methodology, a profound change is needed in order to provide information on the key aspects of the paths to achieving the objectives. There are no details of key elements for validating the study. And also, the authors do not refer any ethical implication of their study.	Thank you for this important observation. We agree that additional procedural details will help others replicate the study. We have revised the methodology section to include specific steps during data collection, including the interview protocol, consent process, and coding procedures.<br><br>We appreciate your concern. While we aimed to balance detail with conciseness, we believe the original version includes sufficient information for replication. However, to enhance clarity, we have added brief clarifications in key areas such as participant recruitment and data coding, without overloading the section with procedural details.

3. Results

**Reviewer Comment**	**Authors' Agreement and Rebuttal Response**
Figure 2 should give percentages rather than numbers	Thank you for noting this. We agree that clearer labeling improves readability and we have revised it.
Socio demographic characteristics of Respondents Why claim a 100% response rate if the number of control cases is not double the number of cases?	We thank the reviewer for this important comment. The 100% response rate refers to the proportion of selected and eligible participants who agreed to participate and completed the interview, not to the ratio of cases to controls. The case-to-control ratio was determined during the study design phase and does not affect the calculation of the response rate. All 300 sampled pregnant

	women (103 cases and 197 controls) participated fully in the study, resulting in a 100% response rate
what is the relationship between unwanted pregnancy and the distance from home to antenatal care services?	We agree with the reviewer that the relationship between distance to antenatal care services and unintended pregnancy should be clearly stated and supported by evidence. In our study, distance from home to the health facility showed a significant association with unintended pregnancy. Women who traveled 4 km or more to reach ANC services had 2.7 times higher odds of experiencing an unintended pregnancy compared to those who traveled less than 4 km (AOR = 2.7; 95% CI: 1.07–8.75; $p < 0.02$). This finding is consistent with existing literature, which suggests that longer distances to health facilities reduce access to family planning services, limit timely contraceptive use, and negatively influence health-seeking behavior, thereby increasing the risk of unintended pregnancy.
In the Bivariate logistic regression was not included all study variables. What criteria and tests did you apply to select the eligible variable?	We agree with the reviewer that the criteria for selecting variables for multivariable logistic regression should be clearly stated. In this study, all independent variables were initially examined using bivariate logistic regression**.** Variables with a p-value < 0.25 in the bivariate analysis were considered eligible and were entered into the multivariable logistic regression model to control for potential confounding effects. This cutoff was chosen in accordance with widely recommended epidemiological modeling practices to avoid excluding potentially important variables at an early stage.
Obstetric and Reproductive Characteristics of	We appreciate the reviewer’s constructive comments and we agree that the

Respondents Why not include the significance test in Table 2, as you did in Table 1 and 3? It would be fine to explore the history of abortion to make sense of what was reported in the introduction. Believe in importance of family planning? I think this type of question would be assessed with an appropriate instrument, not by simply self-report.	inclusion of significance tests in Table 2 enhances consistency and interpretability. Accordingly, appropriate statistical significance tests have now been added to Table 2, similar to Tables 1 and 3.<br><br>“belief in the importance of family planning.” This variable was assessed through self-report, which is a common approach in similar epidemiological studies; however, we recognize that it may be subject to social desirability bias. This limitation has now been explicitly stated in the limitations section of the manuscript.<br><br>Abortion was discussed in the introduction to highlight the consequences of unintended pregnancy, rather than to describe it as a determinant. Although assessing abortion history is important, this variable was intentionally omitted from the final questionnaire after the pretest. During the pretest, pregnant women expressed discomfort in responding to abortion-related questions, and based on expert opinion, the variable was excluded to maintain participants’ comfort and ethical sensitivity
Behavioral characteristics of study participants It is unclear why the p-values for the smoking habit category variables are missing. These variables could be grouped into a single variable to improve comparability across strata.	We thank the reviewer for this insightful comment. The p-values for the smoking habit categories were not initially reported because the categories were presented descriptively and the distribution of smokers across cases and controls was very small, which limited the statistical power for meaningful comparison across multiple categories

4. Discussion

Reviewer Comment	Authors' Agreement and Rebuttal Response
The discussion should be separated into sections according to determinant´s nature.	We appreciated your perspective that separating the discussion into sections based on the determinant's nature is highly effective. It allows readers to follow the argument logically, distinguishing between economic, social, political, or cultural determinants and each determinant can be explored thoroughly without overlap or confusion.
The authors did not refer any study limitations and suggestions for future research direction. there is a chance of self reporting bias and this should be discussed in the discussion section. There is also a chance of misclassification between cases and controls.	We agree with the point raised. The absence of a discussion on study limitations and future research directions weakens the overall rigor of the paper. So that by agreeing with the reviewer Idea we have addressed these issues.
