# Supplementary material for: Determinants of an unintended pregnancy among women attending ante natal care at health facilities in Lemi Kura sub city, Addis Ababa, Ethiopia: Institution based case-control study
Source: PLOS Glob Public Health. 2026 Jul 9;6(7):e0004920. doi: 10.1371/journal.pgph.0004920 (PMC13349197; doi:10.1371/journal.pgph.0004920)
Supplement: Attachment — Submitted filename: Rev 1 - Assoc Editor Comments.pdf [file pgph.0004920.s004.pdf]

PGPH-D-25-01555R1: Determinants of an Unintended Pregnancy among Women Attending Ante Natal Care at Health Facilities in Lemi Kura Sub City, Addis Ababa, Ethiopia: Institution based Case-Control Study

Comments For Authors from the Associate Editor

Introduction

1. “A study conducted by the Guttmacher Institute in Ethiopia reported that …”
    - This is still not clear. 101 per 1000 is 10.1 percent. How does this relate to the 42% and 49%?
2. “Globally, unintended pregnancies account for a substantial proportion of unsafe abortions. Of the estimated 210 million pregnancies …”
    - The cited reference (9) is a study specific to Iran. I suggest you update this literature review with recent global figures from, e.g., https://www.guttmacher.org/fact-sheet/induced-abortion-worldwide.
3. “Alarmingly, 40% of these abortions are performed on women younger than 25 years of age …”
    - The cited reference (10) is a study specific to South West Ethiopia. I cannot find in this paper the quoted figures of 40% of abortions and 68,000 maternal deaths. Is the citation correct?
4. “The burden is particularly severe in developing countries, where maternal and neonatal mortality rates remain disproportionately …”
    - The cited reference (11) is over 15 years old. I suggest you consult recent reports from the WHO, United Nations, UNFPA, UNICEF, Guttmacher Institute, or other similar organizations to obtain up-to-date, globally representative statistics for this type of background information. Many of these agencies publish fact sheets and topic briefs on their websites that might be useful.

Methods and Materials

2.1 Study design and setting

5. “The study was carried out at one private hospital (ICMC) and three public facilities: Abebech Gobena Hospital, Woreda 13 Health Center, and Hidasse Health Center.”
    - Reviewer 2 commented on the selection of hospitals and centers (“Why did you select hospitals and centers knowing that they are …”). I think including your response in the manuscript would be useful to readers (i.e., “We intentionally included both hospitals and health centers … Since our study populations were

those of attending … in all health centers and hospitals in Ethiopia there are abortion care service and family planning service provision units.”). Can you merge it into this paragraph?

Study variables and operational definition

6. “Socio-demographic factors such as Age, marriage, education, religion, ethnicity, income …etc. “
    - You can’t use “etc.”; these need to be enumerated in full e.g., in a table or an appendix, or give a reference to a source that lists them (e.g., the source of the questionnaire you used).

2.3 Data collection tool

7. “Instrument validity was further strengthened through pretesting on 5% of the sample …”
    - It could be interesting to include as an example your decision to remove questions about abortion history based on these pretests. You note this in your response to Reviewer 2 but didn’t add it to the manuscript.

2.5 Data processing and analysis

8. “Variables with < 0.25 in the bivariate analysis …”
    - There’s something missing before the “<” in this sentence.
9. “In the bivariate analysis, variables with (p<0.25) were considered candidates …”
    - This paragraph duplicates some material from the preceding one. I suggest you proof-read and rationalize these two paragraphs.

Results

3.4 Factors affecting an unintended pregnancy

10. “Accordingly, …, as determinants of unintended pregnancy after adjustment for potential confounders”
    - What were the adjustment variables you used in your final multivariable regression? It’s important to document these in their entirety. Please provide an exhaustive list in a table or appendix.
11. “Variables such as age of woman, …”
    - Related to the previous point, terms like “such as” suggest that there are more variables than those you listed. It is clearer to include an exhaustive list of all the variables considered, along with their bivariate regression p-values, in a table or appendix.

All Tables

12. Tables 1, 2, 3, and 4 all have p-value columns.
    - Please ensure that the statistical tests you used to generate these p-values are described somewhere. I think you've done this for Tables 3 and 4 but please ensure all are described. The descriptions could appear in the "Methods" section, the table captions, or elsewhere in the text when the tables are referred to.
