# Supplementary material for: Determinants of an unintended pregnancy among women attending ante natal care at health facilities in Lemi Kura sub city, Addis Ababa, Ethiopia: Institution based case-control study
Source: PLOS Glob Public Health. 2026 Jul 9;6(7):e0004920. doi: 10.1371/journal.pgph.0004920 (PMC13349197; doi:10.1371/journal.pgph.0004920)
Supplement: Attachment — Submitted filename: Response.docx [file pgph.0004920.s006.docx]

Response to associate editor comments:

We sincerely thank the associate editor for your guidance regarding our submission entitled *“Determinants of an Unintended Pregnancy among Women Attending Ante Natal Care at Health Facilities in Lemi Kura Sub City, Addis Ababa, Ethiopia: Institution based Case-Control Study.”*

We have carefully addressed all the requested points:

1. **Tables** – All main tables have been incorporated into the manuscript file as editable, cell‑based objects.
2. **Figures** – Main figures have been uploaded separately in the required formats (.tif/.eps), each under the 10MB size limit.
3. **Ethics Approval Documents** – The ethics approval documents have been re‑uploaded with the file type changed to “Other,” ensuring they remain for internal review only.
4. **Financial Disclosure Statement** – We confirm that the drafted Financial Disclosure statement is complete, accurate, and acceptable for publication alongside our paper.

We appreciate your support and look forward to the continuation of the review process.

Sincerely,

Amideyesus Adinaw

On behalf of all co authors
