# Supplementary material for: Determinants of an unintended pregnancy among women attending ante natal care at health facilities in Lemi Kura sub city, Addis Ababa, Ethiopia: Institution based case-control study
Source: PLOS Glob Public Health. 2026 Jul 9;6(7):e0004920. doi: 10.1371/journal.pgph.0004920 (PMC13349197; doi:10.1371/journal.pgph.0004920)
Supplement: Attachment — Submitted filename: Response to Reviewers.doc [file pgph.0004920.s007.doc]

### Response to Reviewers

**Manuscript ID:** PGPH-D-25-01555R2

**Title:** Determinants of an Unintended Pregnancy among Women Attending Ante Natal Care at Health Facilities in Lemi Kura Sub City, Addis Ababa, Ethiopia: Institution based Case-Control Study

**Journal:** *PLOS Global Public Health*

Dear Dr. Wheldon,

Thank you for the opportunity to submit this revised manuscript. We appreciate your positive feedback and clear guidance on the final two minor matters. We have addressed both comments directly in the text.

Below is our point-by-point response detailing the revisions made.

#### Comment 10: *Adjustment variables in multivariable model... Please add this table to the manuscript and refer to it in the text.*

**Response:** We agree. The appendix table has been integrated into the main text as **Table 4** (*"Variables included in the final multivariable logistic regression model"*). An explicit reference to this table has been added to Section 3.4 (Data Analysis). Subsequent tables have been renumbered accordingly.

#### Comment 11: *use of “such as”... I suggest just deleting the words “such as” to resolve the confusion. The tables listing the variables need to be in the revised manuscript somewhere, and a reference needs to be added to the text.*

**Response:** Thank you for noticing this. Because these lists are exhaustive, we have deleted the phrase “such as” from paragraphs 2 and 3 of Section 3.4 to remove ambiguity. We also added explicit citations pointing readers to the newly integrated **Table 4** at the end of both paragraphs.

### Journal Requirements Checklist

- **References:** The reference list was audited; all citations are complete, correct, and contain no retracted papers.
- **ORCID iDs:** Co-authors have been notified to verify their ORCID iDs in the submission system and except one co-author all co-authors verified well on Zenodo.
- **Figures:** Figures have been processed and validated using the PLOS NAAS tool.

We believe these targeted adjustments fully satisfy the journal's criteria and look forward to your final decision.

Sincerely,

Amideyesus Adinaw Lopiso

On behalf of all coauthors

Email: ebenezerhashbaz@gmail.com
