# Supplementary material for: Determinants of an unintended pregnancy among women attending ante natal care at health facilities in Lemi Kura sub city, Addis Ababa, Ethiopia: Institution based case-control study
Source: PLOS Glob Public Health. 2026 Jul 9;6(7):e0004920. doi: 10.1371/journal.pgph.0004920 (PMC13349197; doi:10.1371/journal.pgph.0004920)
Supplement: Peer review history

# Peer review: Decision Letter 0

**DOI:** 10.1371/journal.pgph.0004920.r001

**Authors:** Mark Wheldon

27 Nov 2025

PGPH-D-25-01555

Determinants of an Unintended Pregnancy among Women Attending Ante Natal Care at Health Facilities in Lemi Kura Sub City, Addis Ababa, Ethiopia: Institution based Case-Control Study

PLOS Global Public Health

Dear Dr. Lopiso,

Thank you for submitting your manuscript to PLOS Global Public Health. After careful consideration, we feel that it has merit but does not fully meet PLOS Global Public Health’s publication criteria as it currently stands. Therefore, we invite you to submit a revised version of the manuscript that addresses the points raised during the review process.

****Please note that Reviewer 2 has submitted additional comments in an attachment in PDF format. You should respond to all comments from the reviewers: those inline at the bottom of this email and those in the attachment. If you cannot access the attachments, please contact the journal.****

Please submit your revised manuscript by Jan 11 2026 11:59PM. If you will need more time than this to complete your revisions, please reply to this message or contact the journal office at globalpubhealth@plos.org. When you're ready to submit your revision, log on to https://www.editorialmanager.com/pgph/ and select the 'Submissions Needing Revision' folder to locate your manuscript file.

Please include the following items when submitting your revised manuscript:

- A rebuttal letter that responds to each point raised by the editor and reviewer(s). You should upload this letter as a separate file labeled 'Response to Reviewers'.
- A marked-up copy of your manuscript that highlights changes made to the original version. You should upload this as a separate file labeled 'Revised Manuscript with Track Changes'.
- An unmarked version of your revised paper without tracked changes. You should upload this as a separate file labeled 'Manuscript'.

Guidelines for resubmitting your figure files are available below the reviewer comments at the end of this letter.

We look forward to receiving your revised manuscript.

Kind regards,

Mark C. Wheldon, Ph.D.

Academic Editor

PLOS Global Public Health

Journal Requirements:

1. Please amend your detailed Financial Disclosure statement. This is published with the article. It must therefore be completed in full sentences and contain the exact wording you wish to be published.

i. Please clarify all sources of financial support for your study. List the grants, grant numbers, and organizations that funded your study, including funding received from your institution. Please note that suppliers of material support, including research materials, should be recognized in the Acknowledgements section rather than in the Financial Disclosure.

ii. State the initials, alongside each funding source, of each author to receive each grant. For example: "This work was supported by the National Institutes of Health (####### to AM; ###### to CJ) and the National Science Foundation (###### to AM)."

iii. State what role the funders took in the study. If the funders had no role in your study, please state: “The funders had no role in study design, data collection and analysis, decision to publish, or preparation of the manuscript.”

iv. If any authors received a salary from any of your funders, please state which authors and which funders.

If you did not receive any funding for this study, please simply state: “The authors received no specific funding for this work.

2. Please ensure that your Ethics Statement is available in its entirety at the beginning of your Methods section, under a subheading 'Ethics Statement'.

3. We ask that a manuscript source file is provided at Revision. Please upload your manuscript file as a .doc, .docx, .rtf or .tex.

4. Please upload separate figure files in .tif or .eps format. Also, remove the figures from your manuscript file but keep the legends.

For more information about figure files please see our guidelines:

https://journals.plos.org/globalpublichealth/s/figures

https://journals.plos.org/globalpublichealth/s/figures#loc-file-requirements

5. In the online submission form, you indicated that “The authors confirm that the raw data supporting the findings of this study are available from the corresponding author upon reasonable request and without unnecessary restriction”.

All PLOS journals now require all data underlying the findings described in their manuscript to be freely available to other researchers, either

1. In a public repository,

2. Within the manuscript itself, or

3. Uploaded as supplementary information.

This policy applies to all data except where public deposition would breach compliance with the protocol approved by your research ethics board. If your data cannot be made publicly available for ethical or legal reasons (e.g., public availability would compromise patient privacy), please explain your reasons by return email and your exemption request will be escalated to the editor for approval. Your exemption request will be handled independently and will not hold up the peer review process, but will need to be resolved should your manuscript be accepted for publication. One of the Editorial team will then be in touch if there are any issues.

If the reviewer comments include a recommendation to cite specific previously published works, please review and evaluate these publications to determine whether they are relevant and should be cited. There is no requirement to cite these works unless the editor has indicated otherwise.

Additional Editor Comments (if provided):

[Note: HTML markup is below. Please do not edit.]

Reviewers' comments:

Reviewer's Responses to Questions

**Comments to the Author**

1. Does this manuscript meet PLOS Global Public Health’s publication criteria? Is the manuscript technically sound, and do the data support the conclusions? The manuscript must describe methodologically and ethically rigorous research with conclusions that are appropriately drawn based on the data presented.

Reviewer #1: Partly

Reviewer #2: Partly

**********

2. Has the statistical analysis been performed appropriately and rigorously?

Reviewer #1: Yes

Reviewer #2: No

**********

3. Have the authors made all data underlying the findings in their manuscript fully available (please refer to the Data Availability Statement at the start of the manuscript PDF file)?

The PLOS Data policy requires authors to make all data underlying the findings described in their manuscript fully available without restriction, with rare exception. The data should be provided as part of the manuscript or its supporting information, or deposited to a public repository. For example, in addition to summary statistics, the data points behind means, medians and variance measures should be available. If there are restrictions on publicly sharing data—e.g. participant privacy or use of data from a third party—those must be specified.

Reviewer #1: Yes

Reviewer #2: No

**********

4. Is the manuscript presented in an intelligible fashion and written in standard English?

PLOS Global Public Health does not copyedit accepted manuscripts, so the language in submitted articles must be clear, correct, and unambiguous. Any typographical or grammatical errors should be corrected at revision, so please note any specific errors here.

Reviewer #1: No

Reviewer #2: No

**********

5. Review Comments to the Author

Please use the space provided to explain your answers to the questions above. You may also include additional comments for the author, including concerns about dual publication, research ethics, or publication ethics. (Please upload your review as an attachment if it exceeds 20,000 characters)

Reviewer #1: The paper is interesting, lots of work has gone into it. Rationale is strong however there are some comments to be addressed. Operational definitions should cover the definitions of all the important variables such as unintended pregnancy, short term and long term contraceptive methods, knowledge of the methods, average knowledge of the methods, method failure etc. Figure 2 should give percentages rather than numbers. Authors have largely used old references , they are suggested to use more recent ones. Sample size calculation is not clear or not clearly written. It is not clear how cases and controls were selected, (the method the authors have used). There is a high chance that with weak definitions of unintended pregnancy and method failure there is a chance of self reporting bias and this should be discussed in the discussion section. There is also a chance of misclassification between cases and controls. Language needs to be tightened and some grammatical errors to be remove.

Reviewer #2: Introduction: The text contains fundamental information for the purpose of the study. However, it needs to update the information, provide context, and improve the writing and structuring of the paragraphs in order to ensure greater textual fluency and comprehension by the readers. Methods and Materials: Regarding methodology, a profound change is needed in order to provide information on the key aspects of the paths to achieving the objectives. There are no details of key elements for validating the study. And also, the authors do not refer any ethical implication of their study. Reuslts: When you compare case with controls, make sense to use a significance test for that. It would be fine to explore the history of abortion to make sense of what was reported in the introduction. In the Bivariate logistic regression was not included all study variables. What criteria and tests did you apply to select the eligible variable? Discussion: A mistake in selecting the study variables for analysis can lead to uncertain discussion and conclusion. In a case-control study, the focus is on prior exposures that influenced the outcome. Therefore, distance to antenatal services is not relevant to this analysis. The authors did not refer any study limitations and suggestions for future research direction.

**********

6. PLOS authors have the option to publish the peer review history of their article (what does this mean?). If published, this will include your full peer review and any attached files.

**Do you want your identity to be public for this peer review?**  If you choose “no”, your identity will remain anonymous but your review may still be made public.

For information about this choice, including consent withdrawal, please see our Privacy Policy.

Reviewer #1: **Yes:**  Sarah Saleem

Reviewer #2: No

**********

[NOTE: If reviewer comments were submitted as an attachment file, they will be attached to this email and accessible via the submission site. Please log into your account, locate the manuscript record, and check for the action link "View Attachments". If this link does not appear, there are no attachment files.]

-->Figure Resubmissions:

While revising your submission, we strongly recommend that you use PLOS’s NAAS tool (https://ngplosjournals.pagemajik.ai/artanalysis) to test your figure files. NAAS can convert your figure files to the TIFF file type and meet basic requirements (such as print size, resolution), or provide you with a report on issues that do not meet our requirements and that NAAS cannot fix.

After uploading your figures to PLOS’s NAAS tool - https://ngplosjournals.pagemajik.ai/artanalysis, NAAS will process the files provided and display the results in the "Uploaded Files" section of the page as the processing is complete. If the uploaded figures meet our requirements (or NAAS is able to fix the files to meet our requirements), the figure will be marked as "fixed" above. If NAAS is unable to fix the files, a red "failed" label will appear above. When NAAS has confirmed that the figure files meet our requirements, please download the file via the download option, and include these NAAS processed figure files when submitting your revised manuscript.

Attachment Submitted filename: Rev_1.pdf

# Author comment: Author response to Decision Letter 1

**DOI:** 10.1371/journal.pgph.0004920.r002

4 Jan 2026

Attachment Submitted filename: Response to Reviewers.pdf

# Editor report: Decision Letter 1

**DOI:** 10.1371/journal.pgph.0004920.r003

**Authors:** Mark Wheldon, Mark Wheldon

10 Feb 2026

PGPH-D-25-01555R1

Determinants of an Unintended Pregnancy among Women Attending Ante Natal Care at Health Facilities in Lemi Kura Sub City, Addis Ababa, Ethiopia: Institution based Case-Control Study

PLOS Global Public Health

Dear Dr. Lopiso,

Thank you for submitting your manuscript to PLOS Global Public Health. After careful consideration, we feel that it has merit but does not fully meet PLOS Global Public Health’s publication criteria as it currently stands. Therefore, we invite you to submit a revised version of the manuscript that addresses the points raised during the review process.

I have reviewed your revised manuscript ('Revision 1') and the comments from the two reviewers. Based on these comments I have summarized the outstanding issues that I would like to ask you to resolve in the attached PDF document 'Rev 1 - Assoc Editor Comments.pdf'. Please see that file for a list of requested revisions and prepare a point-by-point response and a revised manuscript as per the journal's usual requirements. The requests are, in the main, requests to update the cited literature and to provide additional information on methods and results for clarification. I hope you will find them manageable.

Please submit your revised manuscript by Mar 27 2026 11:59PM. If you will need more time than this to complete your revisions, please reply to this message or contact the journal office at globalpubhealth@plos.org. When you're ready to submit your revision, log on to https://www.editorialmanager.com/pgph/ and select the 'Submissions Needing Revision' folder to locate your manuscript file.

Please include the following items when submitting your revised manuscript:

- A letter that responds to each point raised by the editor and reviewer(s). You should upload this letter as a separate file labeled 'Response to Reviewers'.
- A marked-up copy of your manuscript that highlights changes made to the original version. You should upload this as a separate file labeled 'Revised Manuscript with Track Changes'.
- An unmarked version of your revised paper without tracked changes. You should upload this as a separate file labeled 'Manuscript'.

Guidelines for resubmitting your figure files are available below the reviewer comments at the end of this letter.

We look forward to receiving your revised manuscript.

Kind regards,

Mark C. Wheldon, Ph.D.

Academic Editor

PLOS Global Public Health

Journal Requirements:

1. Please provide a detailed online Financial Disclosure statement. This is published with the article. It must therefore be completed in full sentences and contain the exact wording you wish to be published.

a) Please clarify all sources of financial support for your study. List the grants, grant numbers, and organizations that funded your study, including funding received from your institution. Please note that suppliers of material support, including research materials, should be recognized in the Acknowledgements section rather than in the Financial Disclosure.

b) State the initials, alongside each funding source, of each author to receive each grant. For example: “This work was supported by the National Institutes of Health (####### to AM; ###### to CJ) and the National Science Foundation (###### to AM).”

c) State what role the funders took in the study. If the funders had no role in your study, please state: “The funders had no role in study design, data collection and analysis, decision to publish, or preparation of the manuscript.”

For more information, please go to our submission guidelines:

https://journals.plos.org/globalpublichealth/s/submission-guidelines#loc-financial-disclosure-statement

2. Please ensure that the funders and grant numbers match between the Financial Disclosure field and the Funding Information tab in your submission form. Note that the funders must be provided in the same order in both places as well.

3. In the online submission form, you indicated that "The authors confirm that the raw data supporting the findings of this study are available from the corresponding author upon reasonable request and without unnecessary restriction".

All PLOS journals now require all data underlying the findings described in their manuscript to be freely available to other researchers, either

a) In a public repository,

b) Within the manuscript itself, or

d) Uploaded as supplementary information.

This policy applies to all data except where public deposition would breach compliance with the protocol approved by your research ethics board. If your data cannot be made publicly available for ethical or legal reasons (e.g., public availability would compromise patient privacy), please explain your reasons by return email and your exemption request will be escalated to the editor for approval. Your exemption request will be handled independently and will not hold up the peer review process, but will need to be resolved should your manuscript be accepted for publication. One of the Editorial team will then be in touch if there are any issues.

For further assistance, you may go to: http://journals.plos.org/globalpublichealth/s/data-availability

4. Please upload your main article file as a .doc, .docx or .rtf file.

5. Please provide separate main figure files in .tif or .eps format only and ensure that all files are under our size limit of 10MB.

For more information about how to convert your figure files please see our guidelines: https://journals.plos.org/globalpublichealth/s/figures

6. Please ensure that you refer to Figure 1 in your text as, if accepted, production will need this reference to link the reader to the figure.

7. We have noticed that you have uploaded Supporting Information files ("SPSS uninteded pregnancy.sav"), but you have not included a list of legends. Please add a full list of legends for your Supporting Information files before or after the references list.

8. We note that the ethics approval documents should not be published with the paper and are for internal information only. Please change the file type to 'Other' to ensure that they are not published.

9. If the reviewer comments include a recommendation to cite specific previously published works, please review and evaluate these publications to determine whether they are relevant and should be cited. There is no requirement to cite these works unless the editor has indicated otherwise.

Additional Editor Comments (if provided):

Please see the attached file 'Rev 1 - Assoc Editor Comments.pdf' for additional requested revisions.

[Note: HTML markup is below. Please do not edit.]

Reviewers' comments:

[NOTE: If reviewer comments were submitted as an attachment file, they will be attached to this email and accessible via the submission site. Please log into your account, locate the manuscript record, and check for the action link "View Attachments". If this link does not appear, there are no attachment files.]

-->Figure Resubmissions:

While revising your submission, we strongly recommend that you use PLOS’s NAAS tool (https://ngplosjournals.pagemajik.ai/artanalysis) to test your figure files. NAAS can convert your figure files to the TIFF file type and meet basic requirements (such as print size, resolution), or provide you with a report on issues that do not meet our requirements and that NAAS cannot fix.

After uploading your figures to PLOS’s NAAS tool - https://ngplosjournals.pagemajik.ai/artanalysis, NAAS will process the files provided and display the results in the "Uploaded Files" section of the page as the processing is complete. If the uploaded figures meet our requirements (or NAAS is able to fix the files to meet our requirements), the figure will be marked as "fixed" above. If NAAS is unable to fix the files, a red "failed" label will appear above. When NAAS has confirmed that the figure files meet our requirements, please download the file via the download option, and include these NAAS processed figure files when submitting your revised manuscript.

Attachment Submitted filename: Rev 1 - Assoc Editor Comments.pdf

# Author comment: Author response to Decision Letter 2

**DOI:** 10.1371/journal.pgph.0004920.r004

23 May 2026

Attachment Submitted filename: Response.docx

# Editor report: Decision Letter 2

**DOI:** 10.1371/journal.pgph.0004920.r005

**Authors:** Mark Wheldon, Mark Wheldon, Mark Wheldon

1 Jun 2026

PGPH-D-25-01555R2

Determinants of an Unintended Pregnancy among Women Attending Ante Natal Care at Health Facilities in Lemi Kura Sub City, Addis Ababa, Ethiopia: Institution based Case-Control Study

PLOS Global Public Health

Dear Dr. Lopiso,

Thank you for submitting your manuscript to PLOS Global Public Health. After careful consideration, we feel that it has merit but does not fully meet PLOS Global Public Health’s publication criteria as it currently stands. Therefore, we invite you to submit a revised version of the manuscript that addresses the points raised during the review process.

Thank you for addressing most of my comments. There are just two minor matters to clear up related to Comment 10 and Comment 11 in your response letter. Please see below under "Additional Editor Comments".

Please submit your revised manuscript by Jul 01 2026 11:59PM. If you will need more time than this to complete your revisions, please reply to this message or contact the journal office at globalpubhealth@plos.org. When you're ready to submit your revision, log on to https://www.editorialmanager.com/pgph/ and select the 'Submissions Needing Revision' folder to locate your manuscript file.

Please include the following items when submitting your revised manuscript:

- A letter that responds to each point raised by the editor and reviewer(s). You should upload this letter as a separate file labeled 'Response to Reviewers'.
- A marked-up copy of your manuscript that highlights changes made to the original version. You should upload this as a separate file labeled 'Revised Manuscript with Track Changes'.
- An unmarked version of your revised paper without tracked changes. You should upload this as a separate file labeled 'Manuscript'.

Guidelines for resubmitting your figure files are available below the reviewer comments at the end of this letter.

As the corresponding author, your ORCID iD is verified in the submission system and will appear in the published article. PLOS supports the use of ORCID, and we encourage all coauthors to register for an ORCID iD and use it as well. Please encourage your coauthors to verify their ORCID iD within the submission system before final acceptance, as unverified ORCID iDs will not appear in the published article. *Only*  the individual author can complete the verification step; PLOS staff *cannot*  verify ORCID iDs on behalf of authors.

We look forward to receiving your revised manuscript.

Kind regards,

Mark C. Wheldon, Ph.D.

Academic Editor

PLOS Global Public Health

**Journal Requirements:**

If the reviewer comments include a recommendation to cite specific previously published works, please review and evaluate these publications to determine whether they are relevant and should be cited. There is no requirement to cite these works unless the editor has indicated otherwise.

Please review your reference list to ensure that it is complete and correct. If you have cited papers that have been retracted, please include the rationale for doing so in the manuscript text, or remove these references and replace them with relevant current references. Any changes to the reference list should be mentioned in the rebuttal letter that accompanies your revised manuscript. If you need to cite a retracted article, indicate the article’s retracted status in the References list and also include a citation and full reference for the retraction notice.

**Additional Editor Comments:**

Comment 10: Adjustment variables in multivariable model. Response: we have now provided a complete list of all variables included in the final model in a table below. (Appendix: Adjustment Variables in Final Multivariable Regression)

---> Please add this table to the manuscript and refer to it in the text.

Comment 11: use of “such as” Response: we replaced vague wording with a complete and explicit list of variables, supported by a table.

---> In paragraphs 2 and 3 under Section 3.4, the variable lists are still prefixed with “such as”, which implies the list is not exhaustive. However, it looks like they are, in fact, complete because there are 16 and 7 respectively, which matches the counts in paragraph 1. I suggest just deleting the words “such as” to resolve the confusion.

---> The tables listing the variables need to be in the revised manuscript somewhere, and a reference needs to be added to the text. E.g., “see Table 2” (or whatever the appropriate table is).

[Note: HTML markup is below. Please do not edit.]

**Reviewers' comments** :

[NOTE: If reviewer comments were submitted as an attachment file, they will be attached to this email and accessible via the submission site. Please log into your account, locate the manuscript record, and check for the action link "View Attachments". If this link does not appear, there are no attachment files.]

**Figure Resubmissions:**

While revising your submission, we strongly recommend that you use PLOS’s NAAS tool (https://ngplosjournals.pagemajik.ai/artanalysis) to test your figure files. NAAS can convert your figure files to the TIFF file type and meet basic requirements (such as print size, resolution), or provide you with a report on issues that do not meet our requirements and that NAAS cannot fix.

After uploading your figures to PLOS’s NAAS tool - https://ngplosjournals.pagemajik.ai/artanalysis, NAAS will process the files provided and display the results in the "Uploaded Files" section of the page as the processing is complete. If the uploaded figures meet our requirements (or NAAS is able to fix the files to meet our requirements), the figure will be marked as "fixed" above. If NAAS is unable to fix the files, a red "failed" label will appear above. When NAAS has confirmed that the figure files meet our requirements, please download the file via the download option, and include these NAAS processed figure files when submitting your revised manuscript.

# Author comment: Author response to Decision Letter 3

**DOI:** 10.1371/journal.pgph.0004920.r006

14 Jun 2026

Attachment Submitted filename: Response to Reviewers.doc

# Editor report: Decision Letter 3

**DOI:** 10.1371/journal.pgph.0004920.r007

**Authors:** Mark Wheldon, Mark Wheldon, Mark Wheldon, Mark Wheldon

15 Jun 2026

Determinants of an Unintended Pregnancy among Women Attending Ante Natal Care at Health Facilities in Lemi Kura Sub City, Addis Ababa, Ethiopia: Institution based Case-Control Study

PGPH-D-25-01555R3

Dear Mr Lopiso,

We are pleased to inform you that your manuscript 'Determinants of an Unintended Pregnancy among Women Attending Ante Natal Care at Health Facilities in Lemi Kura Sub City, Addis Ababa, Ethiopia: Institution based Case-Control Study' has been provisionally accepted for publication in PLOS Global Public Health.

Before your manuscript can be formally accepted you will need to complete some formatting changes, which you will receive in a follow up email. A member of our team will be in touch with a set of requests.

Please note that your manuscript will not be scheduled for publication until you have made the required changes, so a swift response is appreciated.

IMPORTANT: The editorial review process is now complete. PLOS will only permit corrections to spelling, formatting or significant scientific errors from this point onwards. Requests for major changes, or any which affect the scientific understanding of your work, will cause delays to the publication date of your manuscript.

If your institution or institutions have a press office, please notify them about your upcoming paper to help maximize its impact. If they'll be preparing press materials, please inform our press team as soon as possible -- no later than 48 hours after receiving the formal acceptance. Your manuscript will remain under strict press embargo until 2 pm Eastern Time on the date of publication. For more information, please contact globalpubhealth@plos.org.

Thank you again for supporting Open Access publishing; we are looking forward to publishing your work in PLOS Global Public Health.

Best regards,

Mark C. Wheldon, Ph.D.

Academic Editor

PLOS Global Public Health

***********************************************************

Thank you for addressing my comments.

Reviewer Comments (if any, and for reference):
